# Safety and Efficacy of Finerenone in Diabetic Kidney Disease: A Single-Center Retrospective Real-World Comparative Study of Dosing and Clinical Decision-Making in Taiwan

**DOI:** 10.3390/ph19101539

**Published:** 2026-09-28

**Authors:** Jui-Ting Hsu, Pei-Chen Tseng, Lung-Chih Li, Terry Ting-Yu Chiou, Chien-Hua Chiu, Po-Yen Kuo, Chiang-Chi Huang, Kai-Fan Tsai, Tsuen-Wei Hsu, Po-Jung Wu, Yueh-Ting Lee, Chien-Te Lee, Wen-Chin Lee, Chih-Chao Yang

**Affiliations:** 1 Division of Nephrology, Department of Internal Medicine, Kaohsiung Chang Gung Memorial Hospital, Chang Gung University College of Medicine, Kaohsiung 833401, Taiwan; verybiggest@cgmh.org.tw (J.-T.H.); peitse@cgmh.org.tw (P.-C.T.); r5239@cgmh.org.tw (L.-C.L.); tytc107@gmail.com (T.T.-Y.C.); e22209@cgmh.org.tw (C.-H.C.); b9902050@cgmh.org.tw (P.-Y.K.); a9259@cgmh.org.tw (C.-C.H.); b9302095@cgmh.org.tw (K.-F.T.); scwei@cgmh.org.tw (T.-W.H.); b9302057@cgmh.org.tw (P.-J.W.); yuai@cgmh.org.tw (Y.-T.L.); ctlee33@cgmh.org.tw (C.-T.L.); 2 Division of Nephrology, Kaohsiung Municipal Ta-Tung Hospital, Kaohsiung 801735, Taiwan; 3 Division of Nephrology, Kaohsiung Municipal Fong-Shan Hospital, Kaohsiung 830025, Taiwan

**Keywords:** finerenone, diabetic kidney disease, albuminuria, dosing strategies, non-steroidal mineralocorticoid receptor antagonist, real-world study

## Abstract

**Background**: Finerenone improves cardiorenal outcomes in patients with chronic kidney disease and type 2 diabetes, but its early real-world effect on albuminuria, the relative safety and effectiveness of standard versus reduced-dose regimens, and the clinical factors influencing dose selection remain unclear. **Methods**: This single-center retrospective observational study included 100 patients with diabetic kidney disease: 50 received finerenone plus standard care, and 50 contemporaneous controls received standard care alone. Controls met the same eligibility criteria but were not prescribed finerenone because of physician judgment, patient preference, or financial or reimbursement considerations. UACR, serum creatinine, eGFR, and serum potassium were measured at baseline and after a mean of 28 ± 5 days (range, 21–35 days). Standard-dose finerenone was defined as 10 or 20 mg daily; reduced-dose regimens included alternate-day or intermittent weekly schedules. The principal efficacy analysis used adjusted log-transformed UACR to account for baseline imbalance and skewness. Responder, dose-stratified, post-hoc pairwise, and AIC-based model-selection analyses were exploratory. **Results**: Baseline characteristics were generally balanced, although baseline UACR was significantly higher in the finerenone group. In ANCOVA of log-transformed 4-week UACR adjusted for baseline ln(UACR), eGFR, serum potassium, SGLT2 inhibitor use, and ACEi/ARB therapy, finerenone was associated with lower albuminuria than usual care (adjusted geometric mean ratio, 0.73; 95% CI, 0.56–0.95; *p* = 0.019). The unadjusted absolute UACR change was presented descriptively. A UACR reduction of ≥30% occurred more often with finerenone than with usual care (36% vs. 14%; odds ratio, 3.46; 95% CI, 1.29–9.26; *p* = 0.014). Changes in serum creatinine, eGFR, and serum potassium did not differ significantly between groups. In exploratory dose-stratified analyses, standard-dose finerenone produced a greater UACR reduction than no finerenone; reduced-dose regimens did not differ significantly from no finerenone, and the direct standard- versus reduced-dose comparison did not reach conventional significance. **Conclusions**: Finerenone initiation was associated with lower adjusted albuminuria at four weeks. Four-week UACR assessment may support early monitoring but is not a validated threshold for treatment continuation, discontinuation, or dose escalation. No short-term safety signal was identified, although the study was not powered to assess comparative or long-term safety. Larger prospective studies are needed to determine whether early UACR changes predict long-term cardiorenal benefit.

## 1. Introduction

Diabetic kidney disease (DKD) remains the leading cause of chronic kidney disease (CKD) and end-stage kidney disease worldwide. Despite substantial advances in glycemic control, blood pressure management, and renin–angiotensin system (RAS) inhibition, many patients continue to experience progressive kidney function decline and persistent albuminuria. Albuminuria is not only a marker of glomerular injury but also an established predictor of CKD progression, cardiovascular events, and mortality [1,2,3,4,5,6,7].

Recent therapeutic developments have substantially improved the management of DKD. Sodium–glucose cotransporter-2 (SGLT2) inhibitors and non-steroidal mineralocorticoid receptor antagonists have emerged as important therapies capable of reducing kidney and cardiovascular risk beyond conventional RAS blockade [1,2,3,4,8,9,10]. Among these agents, finerenone is a highly selective non-steroidal mineralocorticoid receptor antagonist that exerts potent anti-inflammatory and anti-fibrotic effects while minimizing off-target hormonal adverse effects [11,12].

The antiproteinuric efficacy of finerenone was initially demonstrated in the phase II ARTS-DN trial, which showed significant dose-dependent reductions in the urinary albumin-to-creatinine ratio (UACR) [13]. However, because the majority of patients enrolled in the ARTS-DN trial presented with microalbuminuria and only mild renal impairment, these findings could not be directly translated to patients at high or very high risk as stratified by the Kidney Disease: Improving Global Outcomes (KDIGO) criteria. Subsequently, the pivotal FIDELIO-DKD and FIGARO-DKD trials, along with the FIDELITY pooled analysis, established the capacity of standard-dose finerenone to reduce kidney disease progression and cardiovascular events across a broad spectrum of chronic kidney disease (CKD) severity, demonstrating early and sustained reductions in albuminuria [14,15,16].

Despite these proven long-term cardiorenal benefits, translating standard-dose regimens into routine clinical practice presents a substantial challenge. Large-scale evidence indicates that the risks of hyperkalemia-related hospitalization and permanent treatment discontinuation significantly increase in patients with diabetic kidney disease (DKD) whose estimated glomerular filtration rate (eGFR) falls below 60 mL/min/1.73 m^2^. This safety concern underscores a critical gap in current real-world practice, where clinicians must carefully balance antiproteinuric efficacy against the heightened risk of adverse potassium events in more advanced stages of kidney disease [17,18,19].

Albuminuria is an established predictor of kidney disease progression, cardiovascular events, and mortality [5,7]. Moreover, early treatment-induced reductions in albuminuria are associated with a lower risk of CKD progression, particularly among patients with elevated baseline albuminuria [20]. Identifying the earliest clinically meaningful response to finerenone is therefore essential to optimize follow-up, support treatment adherence, and personalize clinical decisions. Furthermore, concerns over advanced age, impaired kidney function, hyperkalemia risk, and polypharmacy frequently lead clinicians to prescribe reduced or non-standard finerenone doses. Whether these reduced-dose regimens retain meaningful, short-term antiproteinuric efficacy remains uncertain.

We conducted a pilot exploratory real-world retrospective study to evaluate the temporal pattern of albuminuria reduction following finerenone initiation and to compare the safety and efficacy of standard versus reduced-dose regimens on short-term kidney outcomes. We hypothesized that finerenone would induce a measurable reduction in albuminuria within the first month of treatment. Furthermore, we hypothesized that an initial reduced-dose strategy would safeguard high-risk patients against severe hyperkalemia while preserving clinically meaningful antiproteinuric efficacy in routine practice. Given the retrospective single-center design and the finite study period approved by the Institutional Review Board, this study was intended to generate real-world hypotheses rather than provide definitive evidence.

## 2. Results

### 2.1. Patient Characteristics

A total of 100 patients were included, including 50 patients receiving finerenone and 50 controls. Baseline demographic characteristics were generally balanced between groups. However, baseline UACR was significantly higher in the finerenone group (Table 1).

### 2.2. Finerenone Dosage Categories

Among finerenone-treated patients, 23 (46%) received standard-dose therapy and 27 (54%) received reduced-dose regimens. Reduced-dose regimens included alternate-day administration and intermittent weekly dosing schedules (Table 1).

### 2.3. Albuminuria Response at Four Weeks

Because baseline UACR was significantly higher in the finerenone group and UACR values were markedly skewed, the adjusted log-UACR ANCOVA was considered the principal efficacy analysis. After adjustment for baseline ln(UACR), baseline eGFR, baseline serum potassium, SGLT2 inhibitor use, and ACEi/ARB therapy, finerenone initiation was associated with lower 4-week albuminuria than usual care, with an adjusted geometric mean ratio of 0.73 (95% CI, 0.56–0.95; *p* = 0.019). This estimate corresponds to approximately 27% lower adjusted 4-week UACR in the finerenone group relative to controls.

The unadjusted absolute UACR change was reported as a supportive descriptive analysis and showed a mean change of −245.4 ± 1716.3 mg/g in the finerenone group compared with +327.5 ± 1057.7 mg/g in controls (*p* = 0.047).

A reduction in UACR of at least 30% was observed in 18 of 50 finerenone-treated patients compared with 7 of 50 controls (36% versus 14%; odds ratio, 3.46; 95% CI, 1.29–9.26; *p* = 0.014). Given the small absolute number of responders, this statistically significant result should be interpreted as an exploratory observation requiring validation in larger cohorts.

### 2.4. Renal Function

At four weeks, no significant differences were observed between the finerenone and control groups in serum creatinine (2.09 ± 1.34 mg/dL versus 2.56 ± 2.00 mg/dL, *p* = 0.166) or estimated glomerular filtration rate (44.7 ± 28.9 mL/min/1.73 m^2^ versus 43.3 ± 29.6 mL/min/1.73 m^2^, *p* = 0.812). Within the finerenone group, paired analysis showed that mean eGFR changed from 45.6 ± 28.9 mL/min/1.73 m^2^ at baseline to 44.7 ± 28.9 mL/min/1.73 m^2^ at 4 weeks, corresponding to a modest mean dip of −0.9 mL/min/1.73 m^2^ that was not statistically significant (paired *t*-test, *p* = 0.312). The distribution of eGFR categories was also similar between the two groups. These findings suggest that the antiproteinuric effect of finerenone observed within four weeks was not accompanied by a measurable decline in kidney function during the short observation period (Table 2).

At 4 weeks, systolic blood pressure changed from 140.8 ± 18.8 mmHg to 139.2 ± 17.5 mmHg in the finerenone group (paired comparison, *p* = 0.421) and from 141.4 ± 22.1 mmHg to 140.6 ± 20.3 mmHg in the control group (paired comparison, *p* = 0.654). Between-group changes in systolic and diastolic blood pressure were not statistically significant, consistent with the modest blood-pressure-lowering effect of finerenone reported in prior outcome trials.

### 2.5. Safety

Hyperkalemia was infrequent during the 4-week follow-up: serum potassium ≥5.5 mmol/L occurred in 1 of 50 finerenone-treated patients and 5 of 50 controls, while serum potassium ≥6.0 mmol/L occurred in 0 and 1 patient, respectively. No patient receiving finerenone discontinued therapy because of hyperkalemia. No evident short-term safety signal, including severe hyperkalemia leading to treatment discontinuation or apparent acute kidney injury, was identified during the limited 4-week observation period. However, the modest sample size, non-randomized design, and short follow-up preclude conclusions regarding comparative or long-term safety (Table 2).

### 2.6. Factors Associated with Standard-Dose Finerenone Prescription

Table 3 presents the full multivariable logistic regression model, which included all prespecified candidate clinical variables. In this full model, age, hypertension, and ASCVD were statistically associated with standard-dose finerenone prescription; however, the wide confidence intervals for hypertension and ASCVD indicate considerable imprecision and potential model instability due to the limited sample size and low events-per-variable ratio. The results of the multivariable logistic regression are presented as a forest plot in Figure 1.

To reduce model complexity, an AIC-based backward stepwise selection procedure was additionally performed. The final parsimonious model is presented separately in Table 4 and illustrated in Figure 2. In this exploratory model, older age was associated with lower odds of receiving standard-dose finerenone (OR, 0.93 per 1-year increase; 95% CI, 0.87–0.99; *p* = 0.028), whereas ASCVD (OR, 4.12; 95% CI, 1.18–14.41; *p* = 0.026) and hypertension (OR, 6.85; 95% CI, 1.22–38.45; *p* = 0.029) were associated with higher odds of standard-dose prescription.

### 2.7. Dose-Stratified Analysis

Post-hoc pairwise comparisons showed that the standard-dose finerenone subgroup was associated with a significantly greater relative UACR reduction than the non-finerenone group (mean difference, −68.7%; 95% CI, −118.9% to −18.5%; *p* = 0.004). In contrast, the reduced-dose subgroup was not associated with a statistically significant difference in relative UACR change compared with the non-finerenone group (mean difference, −12.9%; 95% CI, −60.5% to +34.7%; *p* = 0.794). The direct comparison between standard-dose and reduced-dose regimens was borderline and did not reach conventional statistical significance (mean difference, −55.8%; 95% CI, −112.3% to +0.8%; *p* = 0.054). Therefore, these analyses do not establish superiority of standard-dose over reduced-dose regimens and should be interpreted as exploratory (Table 5).

## 3. Discussion

Adjusted ANCOVA provided the principal estimate of the short-term association between finerenone and albuminuria. The model accounted for baseline ln(UACR), eGFR, serum potassium, SGLT2 inhibitor use, and ACEi/ARB therapy, but the observational design does not exclude residual confounding or regression to the mean. The unadjusted absolute UACR comparison is therefore descriptive.

Our findings are broadly consistent with the results of the ARTS-DN, FIDELIO-DKD, FIGARO-DKD, and FIDELITY studies, all of which demonstrated significant reductions in albuminuria and improved cardiorenal outcomes with finerenone [13,14,15,16,21]. The recent CONFIDENCE trial further demonstrated additive early UACR reduction when finerenone was initiated together with an SGLT2 inhibitor, supporting the broader concept of combined cardiorenal risk reduction in albuminuric diabetic kidney disease [22]. Our study extends these observations to routine clinical practice by assessing albuminuria at approximately four weeks, a clinically relevant time point that coincides with the recommended post-initiation laboratory reassessment. In addition, our cohort included patients receiving non-standard alternate-day and intermittent weekly regimens, allowing exploratory comparison with guideline-concordant once-daily dosing. The observation that a measurable antiproteinuric response was detectable within four weeks may be clinically useful because it provides physicians with a practical timeframe for assessing early treatment response.

Beyond albuminuria reduction, finerenone exerts multiple renal protective effects through selective blockade of mineralocorticoid receptor overactivation. Preclinical evidence summarized in prior reviews suggests that finerenone attenuates renal inflammation, oxidative stress, podocyte injury, and tissue fibrosis [11,12]. These mechanisms likely contribute to both short-term reductions in albuminuria and long-term preservation of kidney function observed in outcome trials.

GLP-1 receptor agonists have also emerged as kidney- and cardiovascular-protective therapies in type 2 diabetes and chronic kidney disease, particularly following the FLOW trial. In the present cohort, baseline GLP-1 receptor agonist prescription was minimal (<5%) in both groups, largely reflecting local insurance reimbursement restrictions in Taiwan during the study period, and use did not differ meaningfully between groups [23].

Reduced-dose regimens included every-other-day, twice-weekly, and three-times-weekly schedules. They were pooled because each schedule included too few patients for separate analysis; the grouping does not imply equivalent finerenone exposure.

Reduced or intermittent dosing was not associated with a statistically significant short-term UACR reduction versus no finerenone. This finding is inconclusive because subgroup sizes were small, schedules were heterogeneous, and confounding by indication was possible. The direct comparison with standard dosing was borderline and did not reach conventional statistical significance.

Our real-world findings should also be considered alongside emerging observational data. For example, the Spanish MEDFINE-RWD multicentre study reported substantial 6-month albuminuria reduction and high treatment persistence with finerenone in routine practice. Compared with such European real-world cohorts, our Asian single-center cohort suggests a more cautious prescribing pattern, with frequent off-label reduced or intermittent dosing driven by concerns regarding hyperkalemia, renal vulnerability, advanced age, and medication affordability [24].

Nevertheless, patients with serum potassium ≥4.5 mmol/L or baseline eGFR <45 mL/min/1.73 m^2^ tended to receive reduced-dose regimens. This pattern may reflect clinicians’ concerns regarding kidney safety and hyperkalemia risk when prescribing finerenone in these higher-risk patients.

Assessing UACR approximately four weeks after finerenone initiation may help identify an early treatment-associated change. However, this study does not validate a four-week threshold for continuing, discontinuing, escalating, or modifying therapy. Prospective studies should determine whether early UACR responses can guide individualized treatment or predict longer-term cardiorenal outcomes.

No short-term safety signal was evident, but the study was not designed or powered to establish comparative safety, equivalence, or superiority. Four weeks is also insufficient to characterize long-term hyperkalemia risk, so continued potassium monitoring remains essential [14,17,18,25].

This study has several limitations. Its retrospective single-center design limits causal inference and generalizability, and no prospective analysis plan or a priori power calculation was available. The modest sample, particularly within dose and post-hoc subgroups, limited statistical precision; the low events-per-variable ratio may also have produced model overfitting and wide confidence intervals. Treatment was not randomized, leaving potential confounding by indication. Baseline UACR was higher in the finerenone group, consistent with preferential prescribing to patients with greater albuminuria, but residual confounding, regression to the mean, and baseline skewness may have influenced short-term changes. Heterogeneous reduced-dose schedules were pooled because regimen-specific groups were too small for reliable analysis, and this does not imply pharmacological equivalence. Finally, four weeks was insufficient to evaluate long-term kidney, cardiovascular, or safety outcomes. Although early albuminuria reduction is a meaningful surrogate, it is not a hard kidney outcome [20,26].

## 4. Materials and Methods

### 4.1. Study Design and Setting

This retrospective observational study was conducted at Kaohsiung Chang Gung Memorial Hospital, Taiwan, from April 2024 through July 2025. Patients with diabetic kidney disease were identified from the institutional electronic medical record system.

Controls were contemporaneous patients managed in the outpatient nephrology clinics during the same period. They met the same baseline UACR and eGFR eligibility criteria but were not prescribed finerenone because of physician judgment, patient preference, or financial or reimbursement considerations. Before baseline, all participants received stable standard-of-care therapy, including RAS blockade and SGLT2 inhibitors when clinically appropriate. These therapies were maintained without major dose changes or additions during the four-week observation period.

### 4.2. Inclusion Criteria

Eligible patients were adults aged ≥18 years with type 2 diabetes and diabetic kidney disease, defined by persistent albuminuria and reduced kidney function. Eligibility required baseline UACR > 200 mg/g or eGFR < 60 mL/min/1.73 m^2^; available baseline and approximately four-week measurements of UACR, serum creatinine, eGFR, and serum potassium; and background standard-of-care therapy when clinically appropriate, including RAS blockade and/or an SGLT2 inhibitor unless contraindicated or not tolerated. Patients also could not have a baseline serum potassium concentration that, in routine clinical judgment, precluded finerenone initiation. These criteria identified patients with clinically significant albuminuria or impaired kidney function, consistent with populations in which finerenone has demonstrated cardiorenal benefit.

### 4.3. Study Population

We retrospectively identified potentially eligible patients from outpatient nephrology records during the IRB-approved study period. The final analytic cohort comprised patients with complete paired baseline and 21–35-day follow-up measurements of UACR, serum creatinine/eGFR, and serum potassium. A detailed cohort-assembly diagram is provided in Supplementary Figure S1. Because the source population and reasons for exclusion could not be reconstructed completely from the retrospective record system, the flow diagram presents the final analytic cohort rather than a complete screening cascade.

Most patients had previously received active treatment for albuminuria, including RAS inhibitors and/or SGLT2 inhibitors according to contemporary clinical practice guidelines.

### 4.4. Finerenone Dosing

Standard-dose finerenone was defined as 10 or 20 mg once daily. Regimens below 10 mg daily were classified as reduced dose and included 10 mg every other day, 10 mg three times weekly, 10 mg twice weekly, and 20 mg twice weekly. These intermittent schedules were pooled because each regimen included too few patients for reliable separate analysis; the grouping does not imply pharmacological equivalence.

### 4.5. Data Collection and Outcomes

Baseline demographics, comorbidities, medication use, and laboratory values were extracted from electronic medical records. Laboratory measurements were obtained immediately before baseline or finerenone initiation and again after a mean of 28 ± 5 days (range, 21–35 days). All 100 participants had complete paired data, so analyses used complete cases without imputation.

The primary efficacy outcome was change in UACR from baseline to four weeks. The primary safety outcome was hyperkalemia, defined as serum potassium ≥5.5 mmol/L.

### 4.6. Statistical Analysis

Because the study was retrospective and exploratory, no prospective statistical analysis plan or formal a priori power calculation was available. The principal efficacy analysis used ANCOVA of log-transformed 4-week UACR, adjusted for baseline ln(UACR), eGFR, serum potassium, SGLT2 inhibitor use, and ACEi/ARB therapy. This approach addressed the baseline UACR imbalance and marked skewness. Continuous variables were summarized as mean ± standard deviation or median (interquartile range), and categorical variables as counts and percentages. Normality was assessed with the Shapiro–Wilk test. Between-group comparisons used independent *t*-tests, Mann–Whitney U tests, chi-square tests, or Fisher’s exact tests, as appropriate. Absolute UACR change was summarized descriptively. Responder analyses, three-group comparisons of relative UACR change, Tukey-adjusted pairwise comparisons, and AIC-based backward selection for the parsimonious dose-selection model were exploratory post-hoc analyses intended to characterize response patterns and prescribing practices. Analyses were performed with IBM SPSS Statistics for Windows, version 26.0 (IBM Corp., Armonk, NY, USA). Statistical significance was defined as a two-sided *p* value < 0.05.

## 5. Conclusions

Finerenone initiation was associated with lower adjusted albuminuria at four weeks. Four-week UACR measurement may support early monitoring but is not a validated treatment-decision threshold. Findings for reduced or intermittent dosing were inconclusive because of small, heterogeneous subgroups. No short-term safety signal was identified, although comparative and long-term safety could not be assessed. Larger prospective studies with longer follow-up should confirm these findings and determine whether early UACR reduction predicts sustained kidney protection.

## Figures and Tables

**Figure 1:**
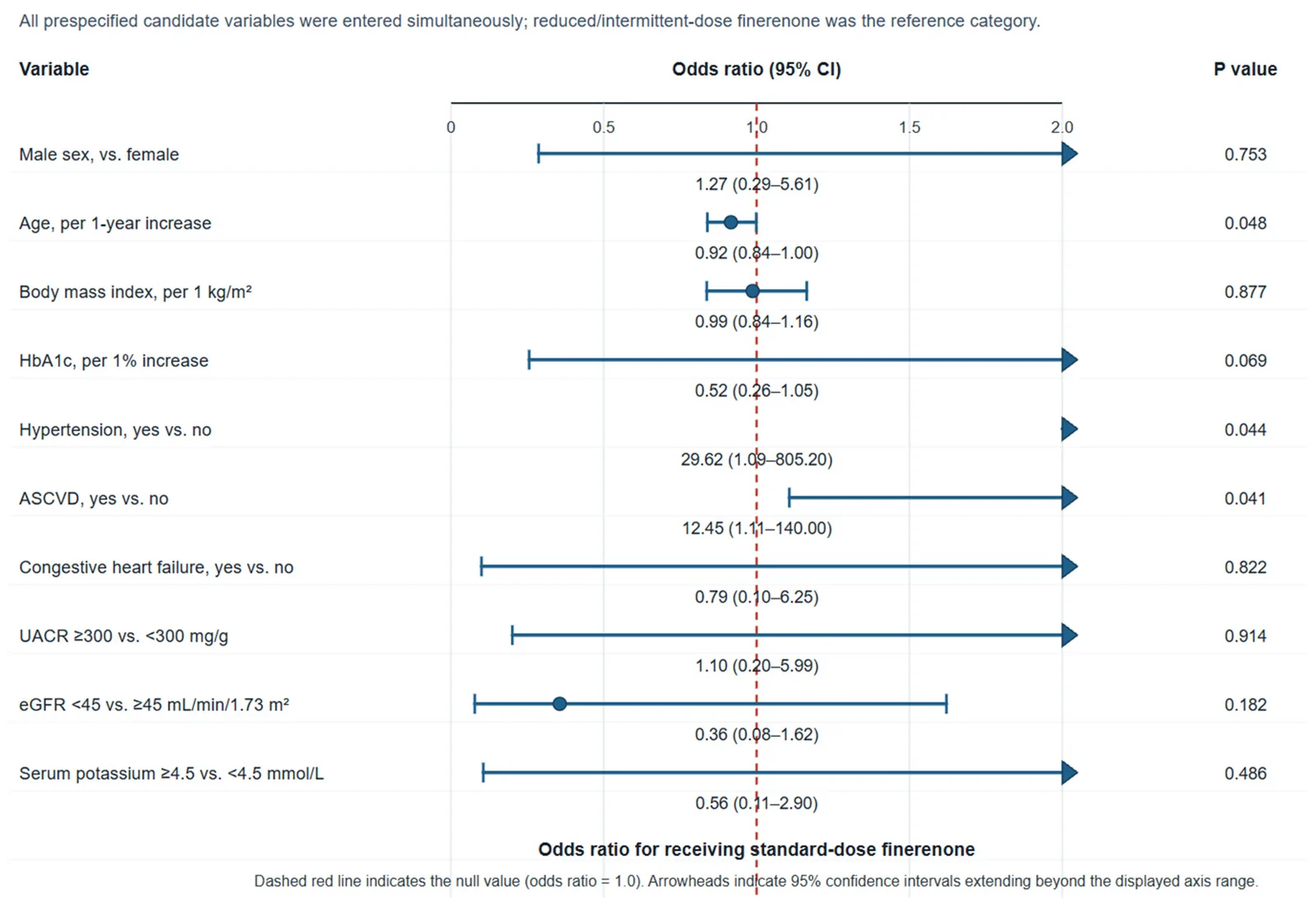
Factors associated with standard-dose finerenone prescription. The dependent variable was prescription of a standard-dose finerenone regimen, defined as 10 or 20 mg once daily, with reduced-dose regimens serving as the reference category. Odds ratios greater than 1 indicate higher odds of standard-dose prescription. Abbreviations: CI, confidence interval; ASCVD, atherosclerotic cardiovascular disease; eGFR, estimated glomerular filtration rate; UACR, urinary albumin-to-creatinine ratio; HTN, hypertension; HbA1c, glycated hemoglobin; K, serum potassium.

**Figure 2:**
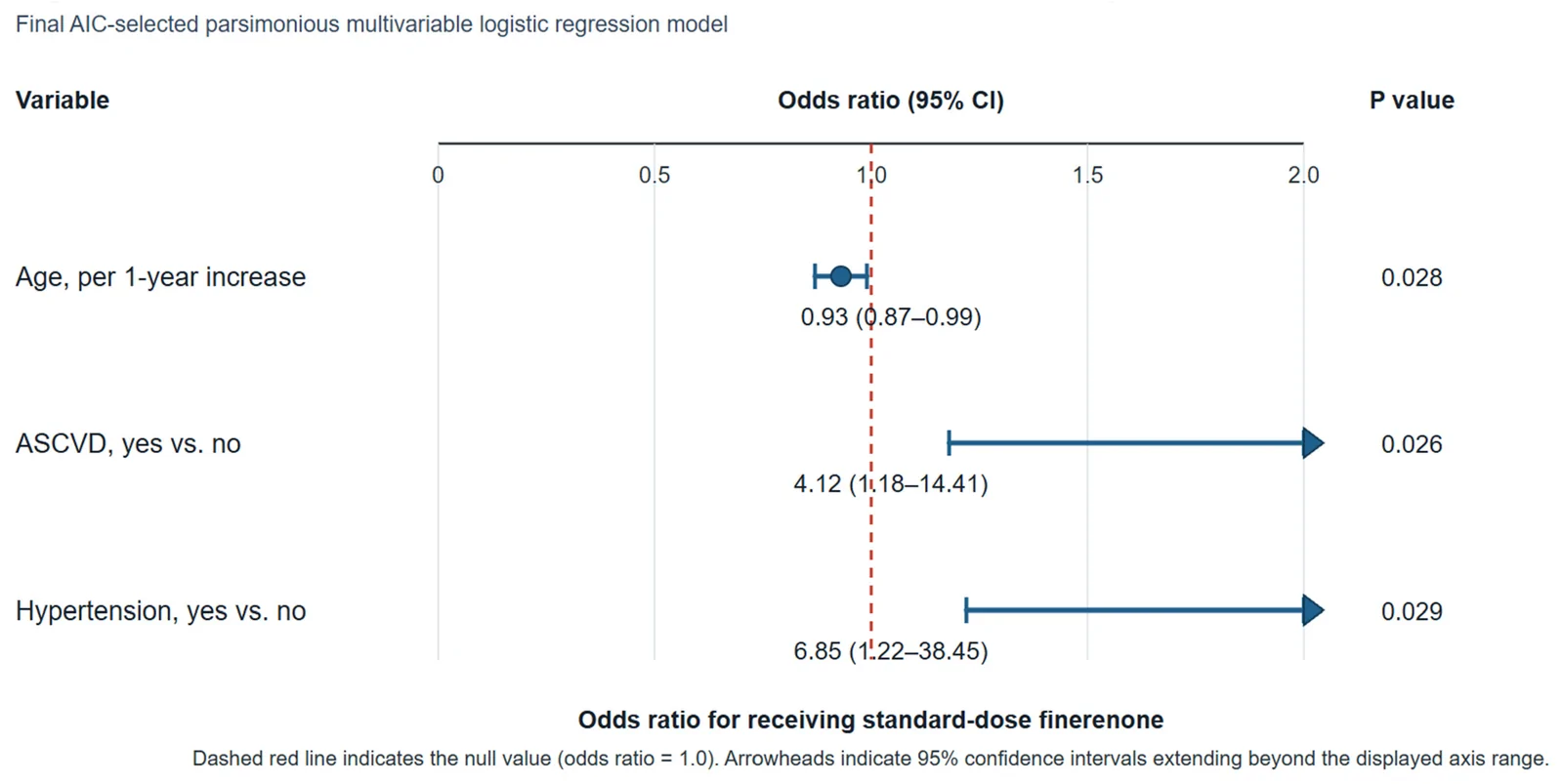
Factors associated with standard-dose finerenone prescription in the final AIC-selected parsimonious multivariable logistic regression model. Figure 2 depicts the final parsimonious model, whereas Table 3 presents the full multivariable model. Odds ratios > 1 indicate higher odds of receiving standard-dose finerenone (10 or 20 mg once daily) relative to reduced/intermittent-dose regimens. Abbreviations: CI, confidence interval; ASCVD, atherosclerotic cardiovascular disease.

**Table 1:** Demographic and Clinical Characteristics of the Participants at Baseline.

Characteristic	Finerenone (n = 50)	Control (n = 50)	Total (n = 100)	*p* Value
Age (years)	66.8 ± 13.5	65.0 ± 9.8	65.9 ± 11.7	0.46
Female Sex	18 (36%)	14 (28%)	32 (32.0%)	0.40
Body-mass index (kg/m^2^)	26.8 ± 4.7	25.7 ± 4.4	26.2 ± 4.5	0.21
Glycated hemoglobin (%)	7.4 ± 1.3	7.1 ± 1.1	7.2 ± 1.2	0.19
eGFR (mL/min/1.73 m^2^)	45.6 ± 28.9	44.6 ± 30.2	45.1 ± 29.4	0.86
≥60	11 (22%)	15 (30%)	26 (26.0%)	
30–59	20 (40%)	15 (30%)	35 (35.0%)	
<30	19 (38%)	20 (40%)	39 (39.0%)	
Median UACR (IQR) (mg/g)	854.2 (247.0–1730.7)	305.0 (98.3–1231.7)	516.7 (161.2–1406.7)	0.03
UACR category (mg/g)				
≦30	0 (%)	0 (%)	0 (0.0%)	
31–299	15 (30%)	25 (50%)	40 (40.0%)	
≧300	35 (70%)	25 (50%)	60 (60.0%)	
Systolic blood pressure (mmHg)	140.8 ± 18.8	141.4 ± 22.1	141.1 ± 20.4	0.88
Serum potassium (mmol/L)	4.3 ± 0.5	4.4 ± 0.7	4.3 ± 0.6	0.40
History of ASCVD	16 (32%)	16 (32%)	32 (32.0%)	1.00
Concomitant medications-no. (%)				
ACEi/ARB	48 (96%)	50 (100%)	98 (98.0%)	0.16
SGLT2-i	32 (64%)	29 (58%)	61 (61.0%)	0.54
Lipid lower Medication	44 (88%)	40 (80%)	84 (84.0%)	0.28
Diuretic	14 (28%)	17 (34%)	31 (31.0%)	0.52
Finerenone doses				
20 mg daily (QD)	3 (6%)			
10 mg daily (QD)	20 (40%)			
10 mg every other day (QOD)	8 (16%)			
10 mg three times a week (TIW)	15 (30%)			
10 mg two times a week (BIW)	3 (6%)			
20 mg two times a week (BIW)	1 (2%)			

Abbreviations: UACR, urinary albumin-to-creatinine ratio; ASCVD, atherosclerotic cardiovascular disease; ACEi, angiotensin-converting enzyme inhibitor; ARB, angiotensin II receptor blocker; QD, once daily; QOD, every other day; BIW, twice weekly; TIW, three times weekly; IQR, interquartile range. Note: Estimated glomerular filtration rate (eGFR) values were automatically calculated by the institutional laboratory electronic medical record system using the CKD-EPI 2021 equation during the study period.

**Table 2:** Patient Data After 4 Weeks.

Characteristic	Finerenone (n = 50)	Control (n = 50)	*p* Value
Serum creatinine, mean ± SD (mg/dL)	2.09 ± 1.34	2.56 ± 2.00	0.166
eGFR, mean ± SD (mL/min/1.73 m^2^)	44.7 ± 28.9	43.3 ± 29.6	0.812
eGFR category, n (%)			
≧60	10 (20%)	15 (30%)	
31–59	20 (40%)	16 (32%)	
≦30	20 (40%)	19 (38%)	
Serum potassium, mean ± SD (mmol/L)	4.32 ± 0.51	4.54 ± 0.66	0.057
[K^+^] <5.5 mmol/L, n (%)	49 (98%)	45 (90%)	
[K^+^] ≧5.5 mmol/L, n (%)	1 (2%)	5 (10%)	
[K^+^] <6.0 mmol/L, n (%)	50 (100%)	49 (98%)	
[K^+^] ≧6.0 mmol/L, n (%)	0 (0%)	1 (2%)	
Absolute change in serum potassium, (mmol/L)	+0.050 ± 0.4	+0.178 ± 0.6	0.209
Urinary albumin-to-creatinine ratio (UACR), mg/g			
Mean ± SD	1391.1 ± 2428.3	1131.2 ± 1495.5	
Median (IQR)	770.1 (245.1–1492.9)	413.6 (104.4–1781.9)	0.521
UACR category, n (%)			
≦30	2 (4%)	3 (6%)	
31–299	14 (28%)	21 (42%)	
≧300	34 (68%)	26 (52%)	
Absolute change in UACR, mean ± SD (mg/g)	−245.4 ± 1716.3	+327.5 ± 1057.7	0.047
UACR reduction ≥30% from baseline, n (%)	18 (36%)	7 (14%)	0.014
Odds ratio (95% CI) for ≧30% reduction	3.46 (1.29–9.26)	—	

Note: Data are presented as mean ± SD, median (IQR), or n (%), as appropriate. Absolute changes were calculated as the 4-week value minus the baseline value. Negative values indicate a decrease from baseline. *p* values compare the finerenone and control groups. Abbreviations: CI, confidence interval; eGFR, estimated glomerular filtration rate; IQR, interquartile range; SD, standard deviation; UACR, urinary albumin-to-creatinine ratio.

**Table 3:** Full Multivariable Logistic Regression Model of Factors Associated with Standard-Dose Finerenone Prescription.

Variable	Odds Ratio	95% CI	*p* Value
Sex (male vs female)	1.269	0.287–5.609	0.753
Age (per 1-year increase)	0.916	0.839–0.999	0.048
Body mass index (per 1 kg/m^2^)	0.987	0.837–1.164	0.877
Glycated hemoglobin (per 1%)	0.519	0.256–1.053	0.069
Hypertension (yes vs. no)	29.619	1.090–805.195	0.044
ASCVD (yes vs. no)	12.448	1.107–139.995	0.041
Congestive heart failure (yes vs. no)	0.789	0.100–6.245	0.822
UACR (≥300 vs. <300 mg/g)	1.098	0.201–5.995	0.914
eGFR (<45 vs. ≥45 mL/min/1.73 m^2^)	0.356	0.078–1.621	0.182
Serum potassium (≥4.5 vs. <4.5 mmol/L)	0.555	0.106–2.899	0.486

Note: This table presents the full multivariable logistic regression model including all candidate clinical variables. Given the modest number of finerenone-treated patients and the limited number of standard-dose events, the events-per-variable ratio was low. The wide confidence intervals, particularly for hypertension and ASCVD, indicate imprecision and potential model instability; therefore, these estimates should be interpreted as exploratory and hypothesis-generating.

**Table 4:** Final AIC-Selected Parsimonious Multivariable Logistic Regression Model of Factors Associated with Standard-Dose Finerenone Prescription.

Variable	Odds Ratio	95% CI	*p* Value
Age, per 1-year increase	0.93	0.87–0.99	0.028
ASCVD, yes vs. no	4.12	1.18–14.41	0.026
Hypertension, yes vs. no	6.85	1.22–38.45	0.029

Note: The final parsimonious model was selected using backward stepwise logistic regression based on the Akaike Information Criterion. Because of the limited sample size and number of dose-group events, this model should be interpreted as exploratory and hypothesis-generating rather than confirmatory. ASCVD, atherosclerotic cardiovascular disease.

**Table 5:** Post-Hoc Pairwise Comparison of Relative Change in UACR (%) from Baseline to 4 Weeks.

Pairwise Comparison	Mean Difference (%)	95% CI	*p* Value
Reduced-dose finerenone vs. Non-finerenone	−12.9	−60.5 to +34.7	0.794
Standard-dose vs. No finerenone	−68.7	−118.9 to −18.5	0.004
Standard-dose vs. Reduced-dose	−55.8	−112.3 to +0.8	0.054

Note: Mean differences in relative UACR change (%), expressed in percentage points, were calculated as Group 1 minus Group 2. A negative value indicates a greater percentage reduction in UACR in the first-listed group relative to the second-listed group. Confidence intervals and *p* values were adjusted using Tukey’s honestly significant difference procedure. Abbreviations: CI, confidence interval; UACR, urinary albumin-to-creatinine ratio.

## Data Availability

The original contributions presented in this study are included in the article/Supplementary Material. Further inquiries can be directed to the corresponding authors.

## Supplementary Materials

The following supporting information can be downloaded at: https://www.mdpi.com/article/10.3390/ph19101539/s1, Supplementary Figure S1. Cohort Assembly, Treatment Classification, and Follow-Up.

## Abbreviations

The following abbreviations are used in this manuscript:

ACEi	Angiotensin-converting enzyme inhibitor
ANOVA	Analysis of variance
ARB	Angiotensin II receptor blocker
ASCVD	Atherosclerotic cardiovascular disease
CI	Confidence interval
CKD	Chronic kidney disease
DKD	Diabetic kidney disease
eGFR	Estimated glomerular filtration rate
IQR	Interquartile range
KDIGO	Kidney Disease: Improving Global Outcomes
MRA	Mineralocorticoid receptor antagonist
OR	Odds ratio
RAS	Renin–angiotensin system
SD	Standard deviation
SGLT2	Sodium–glucose cotransporter 2
UACR	Urinary albumin-to-creatinine ratio
