# Supplementary material for: Safety and Efficacy of Finerenone in Diabetic Kidney Disease: A Single-Center Retrospective Real-World Comparative Study of Dosing and Clinical Decision-Making in Taiwan
Source: Pharmaceuticals (Basel). 2026 Sep 28;19(10):1539. doi: 10.3390/ph19101539 (PMC13708035; doi:10.3390/ph19101539)
Supplement: Supplementary file 1 [file pharmaceuticals-19-01539-s001.zip › pharmaceuticals-4571664-supplementary.pdf]

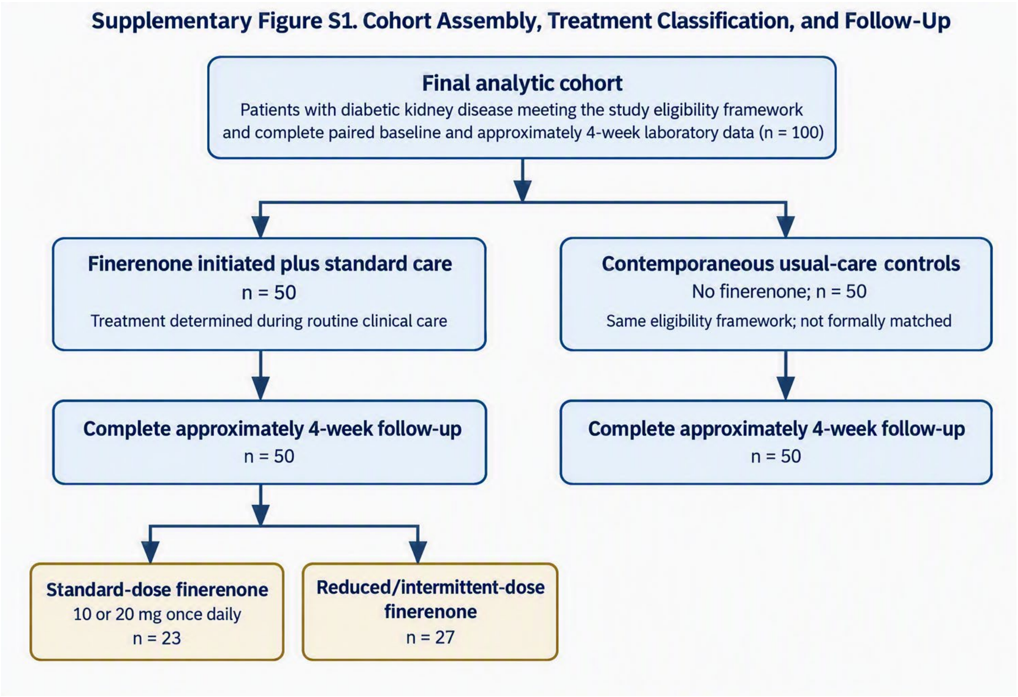

Figure S1. This retrospective comparative study used complete-case analysis of eligible patients with paired baseline and approximately four-week laboratory data. Contemporaneous controls met the same eligibility framework but did not receive finerenone. Controls were not individually or propensity-score matched or formally stratified. Treatment was non-randomized and based on routine clinical decisions; therefore, confounding by indication, residual confounding, and socioeconomic or reimbursement-related selection bias remain possible.
